# Embedded Brain Computer Interface: State-of-the-Art in Research

**DOI:** 10.3390/s21134293

**Published:** 2021-06-23

**Authors:** Kais Belwafi, Sofien Gannouni, Hatim Aboalsamh

**Affiliations:** Department of Computer Science, College of Computer and Information Sciences, King Saud University, Riyadh 11543, Saudi Arabia; gnnosf@ksu.edu.sa (S.G.); hatim@ksu.edu.sa (H.A.)

**Keywords:** electroencephalogram (EEG), EEG signal processing, embedded brain computer interface

## Abstract

There is a wide area of application that uses cerebral activity to restore capabilities for people with severe motor disabilities, and actually the number of such systems keeps growing. Most of the current BCI systems are based on a personal computer. However, there is a tremendous interest in the implementation of BCIs on a portable platform, which has a small size, faster to load, much lower price, lower resources, and lower power consumption than those for full PCs. Depending on the complexity of the signal processing algorithms, it may be more suitable to work with slow processors because there is no need to allow excess capacity of more demanding tasks. So, in this review, we provide an overview of the BCIs development and the current available technology before discussing experimental studies of BCIs.

## 1. Introduction

**Figure 1:**
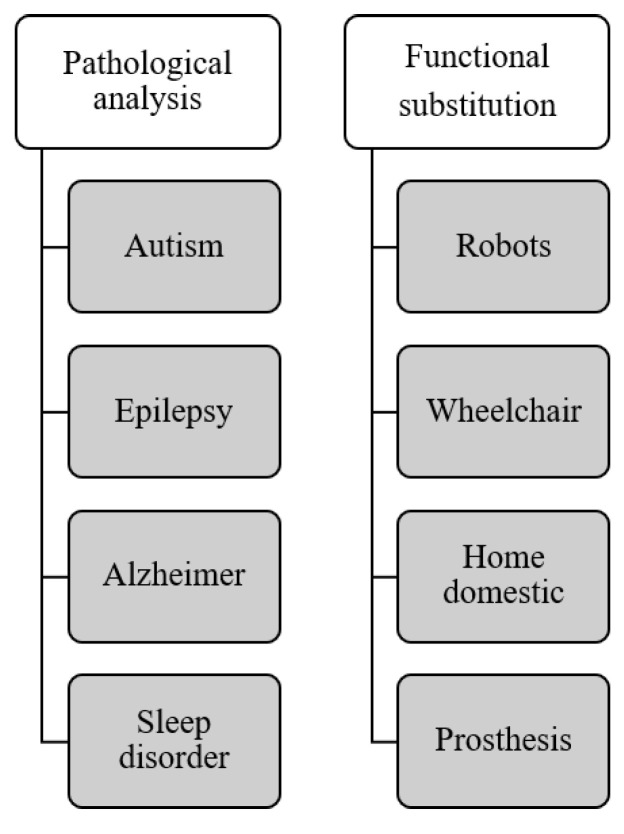
The main application domain of BCI.

In recent years, the advanced potential offers of understanding the physical phenomena that occurred in the brain, as well as the information technologies, make the design of brain computer interface (BCI) systems easier. Non-exhaustively, this technology is mainly used for the functional substitution and pathological analysis. Figure 1 classifies the technologies which were deployed to support people with severe disabilities or doctors into two main categories with examples. There are other application domains including the video games [1,2], the virtual and augmented reality [3,4,5], communication [6,7,8], etc. The focus of this paper is the functional substitution and pathological analysis where the other one are out of the scope of this review.

**Figure 2:**
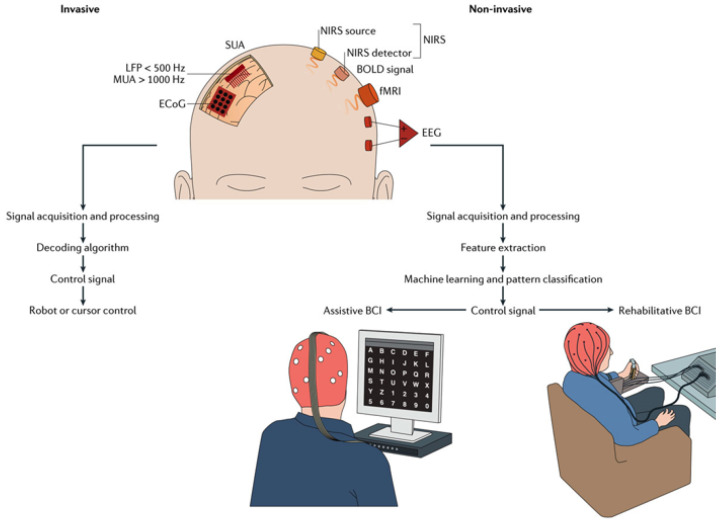
The acquisition techniques [11].

No matter what the application is, the signal processing chain is the same. The BCI system consists of 4 sequential units: (1) signal acquisition, (2) feature extraction, (3) feature translation, and (4) device output [9]. These 4 units are controlled by an operating protocol that defines the onset and timing of operation, the details of signal processing, the nature of the device commands, and the oversight of performance. The first unit recovers the brain signals from the scalp according to invasive or noninvasive methods. As depicted in Figure 2, the invasive technique is always used in a medical pathology analysis. It consists of implementing the electrode inside the brain through a delicate surgical operation. For the other one, it contends to place sensors on the scalp and recovers cerebral activity by measuring the electrical activity (EEG), Functional magnetic resonance imaging (fMRI), and magnetic activity (MEG). The noninvasive technique based on EEG method remains the most solicited for several decades due to its ease of application, low cost, good temporal resolution and not requiring surgical operation [10]. Furthermore, such acquisition systems are based on very accurately analog-to-digital converter (DAC) allowing for the capture of a very small variation in the signal. The second unit allows for increasing signal-to-noise ratio (SNR) of the EEG signal and keeping the pertinent frequency band, depending on the application. The third unit is the feature extraction allowing for reduction in the size of the EEG signals and preparation of the features that are meaningful to the classification stage. Finally, the fourth unit is the classification which permits to discriminate between the class label of the features and convert the labels into logical control signal in order to control the artificial agents or into useful presentation for the practitioner. The common link between these two application classes of BCIs is the measurement of brain activity, where the main difference is about the feedback. In fact, the feedback is sent to the user in the field of functional substitution and for the practitioner in the other one.

Despite the tendency to apply the embedded system technique to BCI, a few embedded BCI (EBCI) systems are presented in literature [12,13]. The most existing BCI platforms are usually implemented in Laptops because it was difficult at the beginning to move the BCI technologies from laboratory scale experiments to the daily life of the people with a neurological disorder [14,15]. Furthermore, the focus of the researchers was to apply these technologies on a personal computer occupied with high computational resources. Such systems are implemented in high performance processors without taking into account the memory resource, power consumption, compactness, the volume, etc. [15,16]. Today, due to the wide spread of the embedded system and the computational resources that can offer, it becomes an emergency to implement the BCI technologies in embedded platforms. So, an EBCI is a microprocessor- or microcontroller-based system of hardware and software. It is designed to analyze the EEG signals and translates them into commands, that are relayed to an output device to carry out a desired action or useful information for practitioner.

Recently, many technical papers have provided short reviews of related work on BCI [11,16,17,18,19,20]. Most, if not all of these reviews, are focused on the application domain of the BCI, the different acquisition technologies, the signal processing algorithm, and limiting the evaluation in order to compare their general advantages/disadvantages, the accuracy of the signal processing, and machine learning algorithms. To support the researchers in the embedded BCI area, as well as other interested parties including EBCI architecture, online/offline BCI, evaluation criteria of the embedded BCI systems, this paper provides a wide and deep look into state-of-the-art contributions in the field of embedded architecture of BCI systems. For this purpose, we followed a systematic approach by addressing each of the following criteria for each reviewed paper.

**Concentration/Stimulation?**

The concentration/stimulation ($C_{s}$) criterion is used to know the type of the signals controlling the system. The existing BCI systems are controlled either by the evoked potential (EP), such as P300, steady state evoked potential (SVEP), or by the spontaneous signals (SP), like motor imagery [21]. Some studies present hybrid systems that combined these two signals to exploit the advantage of each signal type and to get highly accurate system [22]. The most efficient system is the one that does not require the gaze movements and an important effort to command the system [23]. For example, on the one hand, Chabuda et al. proposed an efficient implementation of BCI based on a high-frequency SSVEP. The proposed BCI used a filter block to attenuate all activities which are not phase-locked to the stimulation [24]. Despite the good performance obtained by the proposed system, this last requires the user’s disposition in front of simulation panel and needs an effort of concentration. On the other hand, Belwafi et al. proposed an embedded BCI system allowing people with severe disabilities to control the home domestic by thought using two motor imagery actions [23]. The proposed system allows the user to move freely and interact easily without any constraints. Thus, the BCI system controlled by the evoked potential signal seems to be inappropriate for people with concentration difficulties or with sight problems when the acquisition process becomes unfeasible. Hence, in this evaluation, the system controlled by the spontaneous EEG signals will be seen as a good and an efficient system in contrast to the system controlled by the evoked potential EEG signals.

**Adaptability?**

The EEG signals are always targeted by destructive interferences called artifacts. These artifacts can be categorized into physiological and non-physiological artifacts [25]. On the one hand, the physiological artifacts are mainly generated by the biological activity of the system’s user. For example, these artifacts are generated by the heartbeat, eye movement, muscle activation and user movement [26,27,28]. Fortunately, the methods to eliminate these artifacts are quite simple because they represent repeated morphology waves related to body member function that can be learned by the system during the training phase. On the other hand, the non-physiological artifacts are related to the electrode interface, as well as to the acquisition system and the environment in which it operates. These artifacts manifest in a wide variety of morphologies that mask normal EEG signals and their removal remains a delicate task. The compensation of these artifacts can be done a priori or a posteriori. The first approach allows us to remove the artifacts during the recording process by guiding the patient/user to follow some instructions, such as avoiding eye blinking, minimizing the movements, etc. The second approach allows us to remove the artifacts using digital filtering techniques in order to preserve the useful information and remove artifacts. There are two main approaches to filter the EEG signal: dynamic and static. The dynamic filtering process allows us to remove artifacts carefully in a dynamic manner by taking into consideration the subject’s inter-variability where the static filter applied the same filter parameters for all the users/patients. In this review, a BCI system is classified as a good system when the EEG signal processing algorithm removes artifacts according to the dynamic approach which leads to a quasi-stationary system accuracy for all subjects. According to the previous law, the system presented in Reference [29] is classified as a good because the accuracy is almost the same for all users. On the contrary, the system presented in Reference [30] is classified as a bad system due to the accuracy fluctuation from subject to another.

**Performance?**

In the brain computer interface community, many evaluation metrics are used to measure the performance, such as accuracy of classification, Kappa coefficient, mutual information, information transfer rate (ITR), sensitivity, and specificity [31]. The most common one is the accuracy of the classification which allows for measuring of the number of trials classified correctly of total trials. Consequently, in this paper, we evaluate the existing BCI systems using the obtained accuracy for each subject. The accuracy criterion is the most important because it measures the success degree of the EEG signal processing chain to discriminate or to identify trials. For example, in the literature, the BCI systems that have an accuracy lower than 70% are not acceptable [32], whereas the BCI system that guarantees a classification accuracy more than 75% is considered as a successful or a good one. From this point of view, in the study, an evaluation grid based on the accuracy is defined to compare the existing BCI as presented in Table 1. For example, according to this grid, the system presented in Reference [33] is classified as a good one because its accuracy varied between 86% and 100% for all subject. Furthermore, a P300-based BCI allowing people with severe disabilities to manage electronic devices is proposed in Reference [34]. The presented system fails to classify the trials for some subjects with a classification error of about 74%, although, for another subject, the classification accuracy exceeds 90%. Thus, in these reviews, this system is considered as a poor system due to its uselessness in some cases.

**Power consumption?**

**Figure 3:**
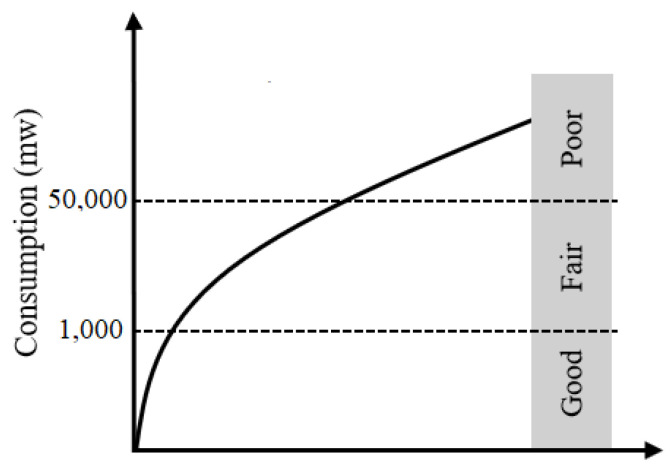
Evaluation grid of the power consumption.

The EEG signal processing algorithms are time consumed due to the huge computation and the traffic load across the whole EBCI system. This matter leads to increase the energy consumption although the EBCI power budget is still confined to a few watts. Hence, the energy consumption criterion is used in order to estimate the amount of the power used by the EBCI system to predict or to translate the brain activities to commands. A consumption grid is defined, as depicted in Figure 3, to classify the EBCI system into three categories. According to this grid, a good EBCI system is the one which consumes less than the other ones where its power consumption does not exceed 1 W. A Fair and a poor system has a power consumption, respectively, less than 5 W and greater than 5 W.

**Online/offline validation?**

International research groups have applied two approaches to validate the BCI system. According to the offline approach, the BCI systems are validated using an existing benchmark. These data are recorded by research groups and shared with the community as a challenge to develop sophisticated signal processing algorithms. For example, the data proposed by the BCI-competition, physionet, etc. Often, the development of any application based on EEG signal processing starts by the validation according to offline approach in order to define the appropriate signal processing techniques. Thereafter, the defined algorithms should be tested with real data according to the online approach to check their effectiveness to extract and classify trials. Frequently, the performance in classifying the EEG trials obtained according to the offline approach decreases significantly compared to the online [35]. For example, the accuracy of the systems presented in References [36,37] decreases, respectively, by 38.8% and 19.8% during the validation of the EEG signal processing chain according to the online approach. To that end, we used online/offline criteria in order to state the presented accuracy with statistical confidence.

The rest of this paper is organized as follows. In Section 2, we evaluate the existing EBCI systems according to the predefined evaluation criteria. We take a closer look into EBCI systems for functional substitution and pathological disorder analysis. In Section 3, we discuss current challenges, the different architecture of EBCI systems, the evaluation criteria of EBCI systems, and possible future research directions on embedded BCI systems. Finally, we provide concluding remarks in Section 4.

## 2. Review of the Embedded BCI Systems

The future of the BCI systems will heavily rely on the computing ecosystem, like any other application domains [12,38]. Today, despite the tremendous interest in implementing the BCI systems into embedded platforms, there are a few embedded BCI (EBCI) systems presented in literature. In fact, at the beginning, the BCI systems are moving slightly from the laboratory to the real world in order to use these systems in the daily life of people with severe disabilities or using them to assist doctors during the analysis of pathological disorders. So, at first, there is a reason in implementing such systems in a personal computer. Today, with the tremendous advancement in the embedded platforms and especially the availability of the open source platform, it becomes too easy to implement complex signal processing algorithms, while maintaining low power consumption, price and resources, etc.

### 2.1. Review of the Acquisition Systems

The BCI systems consist mainly of two parts, signal acquisition and translation. The embedded signal acquisition part contains electrodes, analog circuit and digital system for neurophysiological signal recording and transmission. These systems can be wired or wireless, depending on the type of the connection between the signal acquisition and translation. The wireless EEG acquiring device plays a vital role in the embedded BCI systems, especially with the existence of active dry electrodes allowing a convenient installation and high-fidelity signals. Today, there are many commercial companies producing wireless acquisition systems: G.tec, Emotiv, Open BCI, Neurosky, etc. For example, G.tec company offers a wearable EEG headset for research applications known by G. Nautilus, which integrates 32 analogic-to-digital (ADC) converters that allow for reaching a sampling rate of 1.024 MHz with high resolution about 24 Bit. It comes with flexible cables to configure the EEG electrode positions and connect to a dry and Wet EEG electrodes. G. Nautilus system is not open-source, and it is connected to an embedded platform which remains impossible. Furthermore, the price is very expensive compared to other ones, such as Open BCI Cyton. In fact, the Open BCI Cyton Board is an Arduino-compatible, 8 differential, high gain and low noise channels. It implements the PIC32MX250F128B microcontroller and Texas Instruments ADS1299 ADC. The Open BCI Cyton is compatible with active and passive electrodes and can support until 16 electrodes.

### 2.2. Embedded BCI Systems for Pathological Disorders

In Reference [13], Chin-Teng et al. proposed an EBCI system that can acquire and analyze EEG signals in real-time to monitor human physiological, as well as cognitive states. The system is composed of a four channel physiological acquisition and an amplification unit, a wireless transmission unit, a dual core signal processing unit with multitask scheduling, a sensing real signal display and monitoring unit, and a warning device. The proposed wireless EBCI system is implemented in a dual core DSP and can predict the drowsiness state with an accuracy around 75%. The presented system respects the predefined criteria expect the adaptability ($A_{d}$) criterion and the runtime, which is considered as little bit high. In fact, authors did not take into consideration the inter-subject variability and applied the same signal processing chain for the five subjects which led to a decrease in the accuracy as the case of the 3rd subject where the obtained classification accuracy was about 58%.

Wijesinghe et al. proposed a novel architecture of a generalized platform that provides a set of predefined features and preprocessing steps that can be configured by a user for BCI applications [15]. The presented system is implemented on a Spartan FPGA with a XC3S500E-PQ208 chip. The architecture integrates a power line noise cancellation and baseline removal to enhance the signal-to-noise ratio, while the feature extraction combines linear and nonlinear, univariate and bivariate measures commonly utilized in BCIs. The platform is validated by implementing a seizure detection algorithm on a epileptic seizure detection and it achieved a classification accuracy of over 96%. The advantage of such architecture is that it integrated different features extraction techniques allowing for maximization of the accuracy, while decreasing the runtime and the power consumption and allowing the user to move freely without any constraints.

In Reference [39], to detect and correct seizure, a signal processing algorithms and control circuit for patient monitoring system is presented. The presented device is designed using cyclone III FPGA. The EEG signal is preprocessed using the spline wavelet, to remove the baseline wander signal, and the adaptive threshold and template matching to predict seizure. The seizure detected control signals are generated and the simulator block was activated. The proposed architecture reaches a very good performance in terms of the run-time and power consumption even though the study does not report any evaluation performance in terms of the accuracy.

Table 2 resumes the existing EBCI systems in literature based on a research in PubMed, IEEExplore, ScienceDirect, and Google scholar research web engines. The existing systems are evaluated using the predefined criteria.

### 2.3. Embedded BCI Systems for Functional Substitution

In Reference [14], Lijun et al. proposed an embedded system to control wheelchair merely by thinking. The signal processing algorithm implemented, in iPhone, using a Xcode programming where they are leveraging using the Software Device Kit (SDK). Other EBCI systems are based on a hardware architecture and they reach a very good performance in terms of the power consumption and run-time. For example, Aravind et al. proposed an embedded system that can be used for controlling electrical devices by thinking using EEG signals [40]. The EEG signal processing chain was composed by band-pass finite impulse response filter, wavelet, and Support Vector Machine (SVM). The proposed system was implemented on SPARTAN 6 FPGA Board; unfortunately, there is no system performance reported.

In Reference [47], a pure hardware system based on the FPGA for EEG-MI classification is presented. The EEG signals are processed as a series of multi-channel images in the continuous time domain showing the energy changes in the cerebral cortex during the MI of the subjects. The accuracy in classification reached 80.5% where the presented design was approximately 8 times faster than the PC in terms of the execution time and decreased the power consumption by a factor 5600 compared to a standard PC.

In Reference [43], Sawan et al. proposed an embedded Wireless Recording Systems to measure the brain activity non-invasively and send the recorded data to a host system to apply the signal processing chain. The suggested system improved the mobility of patients and is used by a doctor to predict the start of epilepsy in two patients.

Taehwan et al. proposed a wearable neurofeedback system, which supports mental status monitoring with EEG and transcranial electrical stimulation for neuromodulation [44]. The proposed architecture includes a self configured independent component analysis (ICA), implemented purely in hardware to separate the source at a low power. Based on the predefined criterion, this system can be considered as a good example of the successfully implemented EBCI system in the literature because it takes into consideration the inter-subject variability, running at a low power, and the overall time is reduced by 34% compared to the time without pipelined structure. However, the suggested architecture missed the online validation to check its effectiveness and measure the real classification accuracy.

Kuo-Kai et al. designed a low-cost FPGA-based SSVEP multimedia control system [48]. The proposed system includes a stimulation panel to evoke the subject’s SSVEP signal. Instead of the bulky personal computer with signal processing software, the SSVEP signal processing algorithms are implemented into a cyclone FPGA by hand coding VHSIC hardware description language (VHDL) to accelerate the runtime of the algorithms. Some existing research works are only focused on the implementation of one block of the signal processing chain.

Some studies were only focused on the implementation of very used BCI algorithms. For example, in Reference [49], Plaumbo et al. implemented the spatial filter algorithm, known by ICA technique which is distinguished by its huge time consuming. The ICA algorithm is implemented on a NI CompactRIO embedded system based on an industrial 400 MHz Freescale MPC5200 processor that deterministically executes LabVIEW Real-time applications on the reliable Wind River VxWorks real-time operating system. Hassen et al., in Reference [50], proposed a chip design using a sampling rate conversion system for BCI machine. The proposed chip only allows us to remove the artefacts from the EEG signals without any implementation of the feature extraction and classification algorithms.

Table 3 resumes the existing EBCI systems, for functional substitution, in literature based on a research in PubMed, IEEExplore, ScienceDirect, and Google scholar research web engines. The existing systems are evaluated using the predefined criteria.

### 2.4. Review of the Embedded Architecture of BCI

In this subsection, we only focused on the embedded architecture of the EBCI system independently from the application domain. The existing EBCI systems are implemented according to three alternatives: Software architecture (SW), Hardware architecture (HW), and HW/SW architecture.

#### 2.4.1. EBCI Based on Software Architecture

More than 90% of the existing EBCI systems are implemented as a software code embedded within a microcontroller, such as ARM, Nios, Microblaze, etc.

The EEG signal processing chain is coded using a et al. Level Language (HLL) as C/C++ independently from the architecture of the microcontroller. This approach is widely used because it allows a rapid prototyping within a reasonable time and cost, especially with the spread of the open-source libraries. The EBCI systems based on SW architecture do not have a critical time and a power consumption constraints.

For example, a low-cost FPGA-based BCI speller application is presented in Reference [65]. The proposed system included a data acquisition system and a real-time signal processing chain implemented within a multiprocessor architecture based on Microblaze soft-core. The signal processing chain, integrated Forward Filter and FLDA algorithm, is coded in C/C++ language and implemented within a Xilinx Spartan 3E FPGA board. A master soft-core processor is integrated to manage the communication between the two other slaves, which are allowed, respectively, to filter the EEG signal and control the simulation panel. The P300 enables a user to type without using his hands and to communicate directly with the application and spell out words by merely looking at the screen. The system achieved an accuracy of 65.37%. The presented study missed the architecture evaluation in terms of the runtime and the power consumption which is certainly decreased compared to the power consumption of a standard Laptop.

In Reference [52], an EEG-based smart living environmental control system to auto-adjust the living environment is proposed. The real-time signal processing unit is integrated down-sampling, Hanning window multiplier, short-time FFT, normalization, moving average, ICA decomposition and drowsy state estimator, etc. These algorithms are implemented on a dual-core processor within OMAP1510 platform. The signal processing unit is coded in C/C++ language and includes an embedded task management algorithm called the multi-task scheduling mechanism, which allows for the management tasks and ensures the accurate sampling rate for EEG signal acquisition and data process. The average accuracy of the proposed system reached 78%. The system took about 2 s to estimate the physiological state and consumed about 1 W.

#### 2.4.2. EBCI Based on Hardware Architecture

EBCI system based on HW architecture are implemented on Field-Programmable Gate Arrays (FPGAs) using the Hardware Description Language (HDL) at the register transfer level to get the complete EEG-based signal processing. In this case, the EEG signal processing chain is exported as an Intellectual Properties (IP) coded in Verilog or Very High Description Language (VHDL). The HW architecture remains the appropriate approach when the timing constraints and the power budget are very stringent. The prototyping time of such architecture is highly important, and the cost of the design complexity and the design time is significantly increased in comparison with the SW architecture.

For example, in Reference [39], Tamilarasi and Sundararajan proposed an EBCI system to detect and cure seizure automatically without physician intervention. The proposed system is implemented in Cyclone III/II development kits. A hardware IP is developed to remove the baseline and the artefacts from the acquired EEG signal. An adaptive thresholding technique is implemented to detect the seizure and generate control signals to activate the stimulator block. The suggested platform achieved a very good performance in terms of processing time and power consumption which are, respectively, ∼120 ns and ∼610 mw. The obtained performances remain discussable due to the miss of the evaluation in terms of the accuracy of the EEG signal processing chain.

In Reference [45], a low-power SoC is presented for the continuous detection of seizure onset in epilepsy patients. The proposed system continuously acquires the EEG signal from 18 electrodes and extracts the EEG features before sending them wirelessly to a central device to predict the start of the seizure via a machine-learning classifier. The feature extraction is done in the SoC to reduce 14× the power consumption by reducing the rate of wireless EEG data transmission. The SoC consumes approximately 9 J per feature vector per one channel. Although the extracted features are classified in a standard laptop which is a power consuming, the proposed system achieved a very good performance in terms of the classification accuracy, the power consumption and even in c time consumption. These performances can be easily enhanced by implementing the classification block based on an embedded processor, which allows patients to move freely without any constraints.

#### 2.4.3. EBCI Based on Hardware/Software Architecture

The HW/SW architecture consists of combining both hardware and software components with respect to the embedded system and operating a co-simulation around the FPGA-based processor architecture to meet the timing constraints, which cannot be satisfied by the software solution. According to this design methodology, the critical blocks of the EEG signal processing chain are implemented using the HDL languages and the non-critical blocks are kept in HLL language. This approach allows us to get more flexibility, while it decreases the EEG signal processing run-time, and even the power consumption.

For example, in Reference [23], Belwafi et al. proposed an efficient HW/SW architecture integrating the entire EEG signal processing chain. In this EBCI system, the artefacts removal block, which is time consuming, is exported as hardware accelerators. The remaining blocks are developed as an embedded software running on an embedded Nios-II soft-core processor. The same team proposed a HW/SW architecture to detect motor imagery signal using a dynamic filter based on WOLA technique [64]. The EBCI system performs fast classification within a time delay of 0.430 s/trial, achieving an average accuracy of 76.80% according to an offline approach and 80.25% using our own recording. The estimated power consumption of the proposed architecture is approximately 0.7 W.

## 3. Challenges and Future Research Directions for EBCI Systems

**Figure 4:**
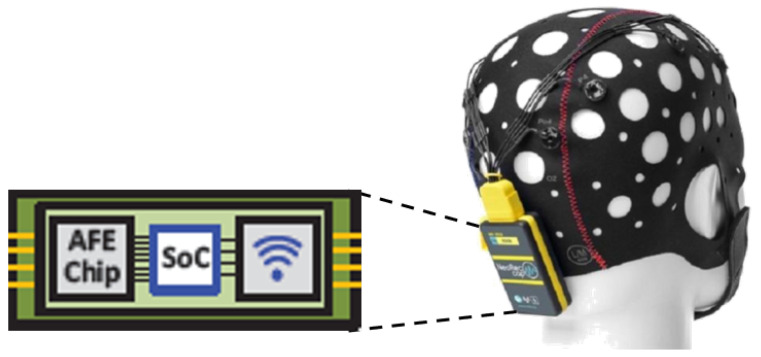
The main application domain of BCI [11].

Our vision of BCI developments that might emerge in the next 10–20 years includes a fully customized, low power, and real-time embedded BCI system. The ECBI system will be mounted on the scalp allowing in the same time to acquire the EEG signal, derived from thousands of neurons, process, and to analyze the brain activities in order to send the decision to the practitioner or the EBCI’s user. Figure 4 shows an overview of the future of the BCI systems.

The advancement in the development of the acquisition BCI, such as NAUTILUS PRO from G.tech company, OpenBCI, and Emotive headset, allows us today to acquire the EEG signals using dry electrodes and send them wireless to a base station for further processing. In Reference [22], Rifai et al. proposed a hybrid BCI which senses a combination classification of mental task, SSVEP, and eyes closed detection using two EEG channels. The brain activities are acquired, through an embedded platform based on Atmega128 microcontroller, and sent to a computer to treat and classify them. The vision of the future BCI systems becomes too easy today, mainly with the existence of the diversity open-source library that allow for easy implementation of the EEG signal processing algorithms. In this respect, as any other expanding domains, as well as with the existing convenient infrastructures, the focus of the BCI community will go in the coming two decades to the embedded implementation of the BCI systems.

The embedded implementation of the BCI systems surely will go faster with the advancement and the capabilities of the existing embedded platforms. A first proof of concept prototype is usually validated on a desktop computer, possibly using a programming language with features that facilitates early prototyping, such as MATLAB, and relies on the emulation of external interfaces [82]. If this initial prototype does not meet its functional requirements, the developer must iterate and modify the application, possibly changing its data types, applying code transformations, and if any refactoring codes to meet the desired requirements. This process is guided by developers knowledge about the impact of these modifications on the final embedded BCI version. Once the prototype is validated on a Desktop computer, the embedded implementation becomes much easier and it remains to explore or identify the appropriate architecture of the EBCI system. In order to simplify this review, we have divided the embedded implementation of the BCI system according to three criteria which represent the keywords to select the appropriate architecture of the EBCI systems.

Some considerations about the limitations and challenges related to the BCI usage and applications are revealed even before moving to the embedded implementation of BCI technology [10,83]. Such limitations we found are:

- Inaccuracy of the BCI in terms of prediction or classifying brain signals.
- The artifacts and outliers that can limit its usability and the interpretability of the extracted features which can be noise-affected due to the low signal-to-noise ratio characterizing the EEG signals.
- Limited ability to read brain signals for those BCIs placed outside the skull.
- Number of ethical issues due to reading people’s inner thoughts [83].
- The security of personal data not being guaranteed against attackers or intruders [83].
- In some cases, requirement for drastic surgery.

### 3.1. Pure Software Architecture

**Figure 5:**
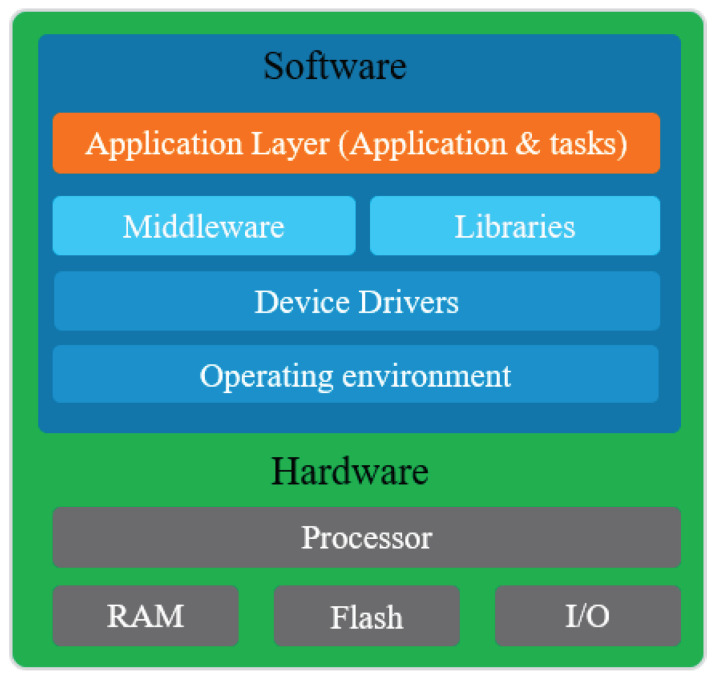
Layers of the EBCI based on software architecture.

This alternative defines the software architecture as a high level abstraction with an embedded infrastructure within which BCI application is deployed and executed [84]. For example, in Reference [42], an embedded BCI system is proposed for driver drowsiness detection. The EEG signal processing algorithm are coded using C/C++ language and implemented within STM32 platform based on an embedded ARM processor. In Reference [14], an EBCI system is proposed based on iPhone to control a wheelchair. An iOS application is developed and integrated with the iPhone to process the EEG signal and generate the control signals of the wheelchair. In this respect, the EBCI system can be a pure software architecture but implemented on an embedded platform. Figure 5 shows the general layers of the EBCI system based on a software architecture. The components at the lowest layers talk directly to the hardware. The top-most layer is aptly called the application layer as it implements the device’s highest level logic and glues together the rest of the components and layers. This solution remains the appropriate alternative for the EBCI systems that do not have timing constraints. Furthermore, the pure software architecture allows a rapid virtual prototyping and implements the application within a reasonable time and cost [64].

The EEG signal processing requires advanced algorithms to filter, extract, and classify trials, where often these algorithms are based on complex mathematical operations. For this reason, at the beginning, the implementation of the EBCI system based on a pure software architecture, within an embedded microcontroller, was too hard and required a huge time to propose a virtual prototype of BCI applications. Today, by dint of the spread of the open source library, it becomes easy to design and deploy EEG signal processing, feature extraction techniques, and intelligent machine-learned onto resource constrained platforms and small single board computers, like FPGA, Raspberry Pi, Arduino, etc. The deployed signal processing chain run locally, without requiring a network connection and without relying on servers in the cloud. As an example of open-source libraries, we found GNU Scientific Library (GSL), CBLAS, ATLAS, OpenCL, OpenCV, SHARK, MLPACK, etc. For instance, Shark is a fast modular library, and it has overwhelming support for supervised learning algorithms, such as linear regression, neural networks, clustering, k-means, etc. It also includes the functionality of linear algebra and numerical optimization. These are key mathematical functions or areas that are very important when performing the EEG signals processing. The prototype and data definitions of these functions are present in their respective header files. To use these functions, it obviously requires compilation and inclusion of the header file in the EEG signals processing chain.

The EEG signal processing chain is based on a deep learning or pattern recognition algorithms. These algorithms are fields with intense computational requirements. Using the software architecture, the EEG chain can be implemented on GPU occupied with Tensor Cores. There are now enough cheap and embedded GPUs that almost everyone can afford a GPU with Tensor Cores in an embedded platform. For example, there are many embedded platforms occupied with GPU, such as Nvidia Jetson Xavier, Jetson Nano, Jetson TX2, NVIDIA Clara AGX, etc. The GPU occupied with tensor flow can be a good choice to implement EEG signal processing chain which is mainly based on matrices computation. In fact, the tensor cores reduce the used cycles needed for the calculation, multiplication, and addition operations, 16-fold in my example, for a 32 × 32 matrix, from 128 cycles to 8 cycles. Furthermore, tensor cores reduce the reliance on repetitive shared memory access, thus saving additional cycles for memory access. The prototyping time of the EBCI based on GPU is reasonable compared to another alternative due to the offered Deep learning frameworks, such as MXNet, PyTorch, TensorFlow, while others rely on GPU accelerated libraries, such as cuDNN, NCCL, and DALI. The EBCI system based on GPU is very powerful and will surely enhance the consuming time but the power consumption of these platforms is too high and can reach 350 W.

### 3.2. Hardware/Software Architecture

**Figure 6:**
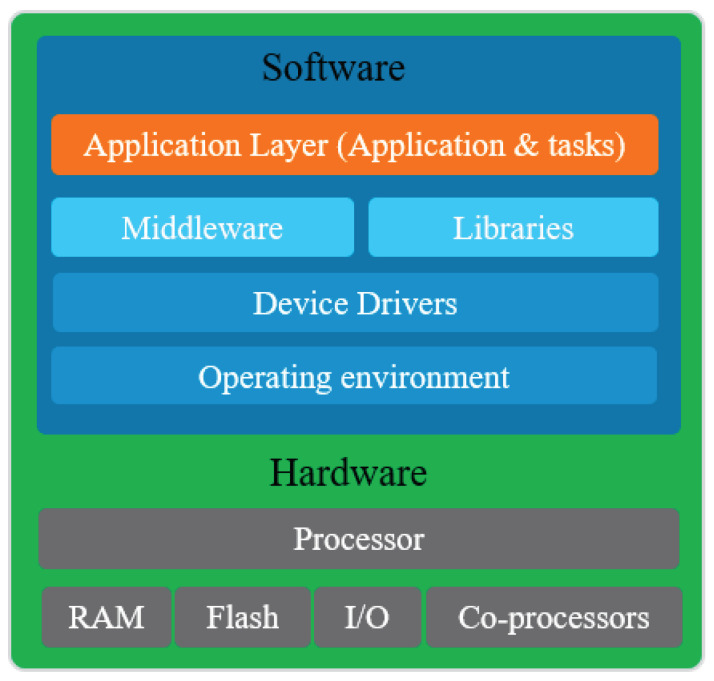
Layers of the EBCI based on hardware/software architecture.

Co-design, HW/SW architecture, is based on the system specification, architectural design, hardware, and software partition. The hardware and software components are combined with respect to the embedded system and are operating a co-simulation around the FPGA-based processor architecture to meet the timing constraints, which cannot be satisfied with the software architecture. In this case, the critical function is exported as a custom logic instruction approach and accelerator co-processor. Figure 6 shows the general layers of the EBCI system based on hardware/software architecture. The components at the lowest layers talk directly to the hardware. The top most layer is aptly called the application layer as it implements the device’s highest level logic and glues together the rest of the components and layers. The architecture of this solution is almost the same as the previous one where the main difference is at the hardware layers which integrates new IPs core. These cores implement the critical parts of the software layers, while profiting from the advantages of the hardware implementation, which executes many instructions in one clock signal instead of executing one instruction in one clock signal as the case of the processor. Using this design methodology, the timing constraint will surely be improved even the power consumption within an increase in the design complexity.

**Figure 7:**
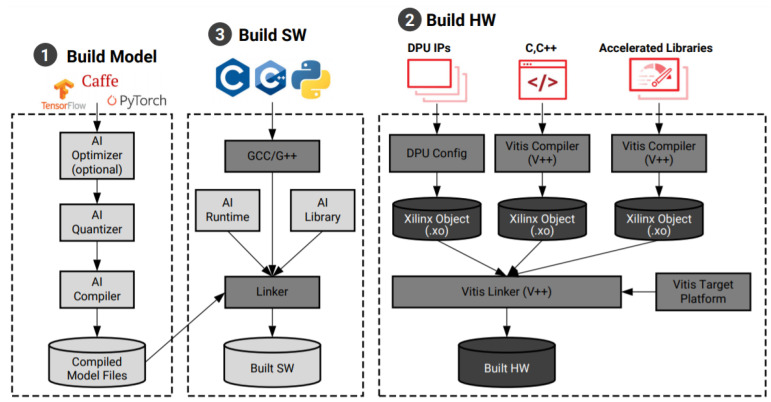
EBCI flow based on Xilinx platforms [85].

The HW/SW architecture also offers the possibility us to use an open-source library, such as the OpenCL, OpenCV, CBLAS, ATLAS, SHARK, MLPACK, etc. This architecture allows us to get at the same time high throughput and low latency. In fact, it allows us to achieve throughput using low-batch size and processes each input as soon as it is ready, resulting in low latency in contrast to the pure software architecture which allows us to get high throughput OR low latency. The pure SW architecture achieves throughput using low-batch size and processes each input as soon as it is ready, resulting in low latency. For example, Xilinx company offers the Vitis AI development environment allowing the acceleration of AI inference on Xilinx hardware platforms. It consists of optimized IP cores, tools, libraries, models, and example designs. It is designed with a high efficiency and ease of use in mind to unleash the full potential of AI acceleration on Xilinx FPGAs and on adaptive compute acceleration platforms (ACAPs). Figure 7 shows the flow to build and design an EBCI system based on Xilinx platform according to the HW/SW architecture. First, the Vitis AI toolchain is used to build the model in the host machine using the caffe, Pytorch, and TensorFlow library. Second, a custom hardware platform is built using the Vitis software platform to include the deep learning processing Unit IP and other kernels. In the Vitis AI release package, pre-built SD card images and Alve shells are included for a quick start and an application development. Finally, developers can build executable software which runs on the built hardware. The applications can be coded with C++ or Python, which calls the Vitis AI Runtime and Vitis AI Library to load and run the compiled model files.

### 3.3. Pure Hardware Architecture

Building an EBCI based on hardware architecture is a challenging task. Here, the key design challenge is to build an extremely powerful system (in terms of the features provided by the system so as to make it easy to use for EBCI users) at a very low power consumption, a low EBCI cost and at the same time meeting all the timing constraints [86]. This alternative consists of implementing the complete EEG signal processing chain within an FPGA using the Hardware description language (HDL) which is a specialized computer language used to program electronic and digital logic circuits. The three common HDLs are Verilog, VHDL, and SystemC. This approach can be used when the timing constraints are very stringent, and it is impossible to respect it with the pure software architecture. The time to obtain the prototype is highly important, and the cost of the design complexity and the design time is significantly increased in comparison with the two previous alternatives.

In many ways, the choice of an application framework for use in an embedded platform is the most important design decision, at least in the case of the embedded systems with human users, such as mobile handsets. In fact, most contemporary users seem to exhibit strong opinions about which application environment their chosen handset supports.

### 3.4. Evaluation Criteria of the Future EBCI Systems

In addition to guidelines, our survey also enabled us to identify a common classification criterion that must be used to compare the EBCI systems. The suggested evaluation criterion takes into consideration the suggested criteria defined in the introduction. So far, the majority of the BCIs has been evaluated based on the accuracy criteria, which computes the percentage of the trial classified correctly [31]. Information Transfer Rate (ITR) is a general evaluation metric devised for BCI systems that determines the amount of information that is conveyed by a system’s output [87].

(1) $$ITR=L[plog_{2}\left(p\right)+log_{2}\left(N\right)+\left(1-p\right)log_{2}\left(\frac{1-p}{N-1}\right)],$$

where *L* is the number of decisions per minute, and *p* is the accuracy of the decision made for the *N* targets. The ITR is the appropriate criterion in the context of the evaluation of the EBCI systems. It will be more suitable to add the criteria: Concentration ($C_{s}$), adaptability ($A_{d}$), power consumption ($P_{w}$), and the offline/online (*O*) in the ITR equation, where the main objective is to easily compare between the future EBCI systems. The $E_{c}$ is suggested to evaluate the EBCI systems in the future by taking into consideration the predefined criteria. Thus, the $E_{c}$ represents the average bits of information contained in each selection.

(2) $$E_{c}=\alpha \beta \gamma \frac{ITR}{\frac{P_{w}}{N_{ch}}}.$$

Table 4 resumes the parameters of the the Equation (2). The weights of α, β, and γ are defined logically in order to establish a comparison grid. The number of channels $N_{ch}$ is integrated in the $E_{c}$ evaluation criteria because this factor is too important, it differs from one study to another, and has a significant weight in the power consumption of the EBCI systems. An EBCI system outperforms better than other ones when it reaching a greater value of $E_{c}$.

## 4. Conclusions

This paper is an attempt to summarize the last two decades of embedded Brain-Computer Interface mostly because of the electroencephalography influence on these systems. Numerous noninvasive EBCIs have been developed, described, and tested. Noninvasive nature of the EEG-based BCIs made them the most popular BCI systems. Their main purpose was to assist practitioner to analyze some pathological disorders and to enable direct non-muscular communication for people with severe disabilities. This resulted in issuing various inexpensive, consumer-grade headsets. Their application potential is vast and ranges from clinical to home-entertainment applications. In this paper, we have delimited our review in the application related to the functional substitution and pathological disorder analysis only.

Six important criteria are defined to evaluate the existing EBCI systems and are represented as a unified ruler applied during our evaluation. The adaptability criterion allows for identification of the EBCI systems that have taken into consideration the inter-subject variability. Concentration/simulation criteria permit to identify the more comfortable EBCI systems. The runtime and the power consumption reflect the performance of the used embedded platform. The last criterion is the validation approach which gives a glance about the stability of the EBCI system and its use in the daily life. The existing EBCI systems are evaluated according to these criteria and allow us to define a few indexes to estimate the EBCI technology’s performance. Furthermore, we have discussed in the future trends the design of the EBCI systems and the limitation and challenges related to the design of EBCI.

In the future, we plan to perform a quantitative analysis of the optimization approaches for EBCI system using machine learning and deep learning algorithms.

The following abbreviations are used in this manuscript:

## Figures and Tables

**Table 1:** Evaluation grid of the accuracy.

Classification Accuracy	Accord
<50	Poor
50–75%	Fair
75–100%	Good

**Table 2:** Summary of related works on embedded BCIs for pathological disorders: N/I: No indication, 0: bad, 1: good.

Work	Year	$C_{s}$	$A_{d}$	Algorithms	Accuracy (%)	Platform	Time (ms)	Power (W)	Online/Offline
[40]	2008	1	0	Hamming window, STFT, PCA, Linear regression	74.6	DSP, ARM processor	42	∼1	Online
[22]	2017	1	0	PSD, ANN	70	Atmega128, AD8553	4000	∼0.9	Online
[41]	2017	1	0	PSD, RMS, Threshold	85	ADS1298, STM32F407vgt6	0.7	∼0.091	Online
[42]	2018	1	0	LP IIR, FFT, SVM	96	STM32F103CB, LMC6464, L3G4200D,	NA	9	Online
[43]	2013	0	1	N/I	N/I	Zarlink ZL70102, MSP430	N/I	2.2	Online
[44]	2014	1	1	ICA, FFT, SVM	91	TI CC2564, FPGA,	N/I	0.45	Offline
[45]	2010	1	1	Bandpass filter, FFT, SVM	93	SoC	6700 ± 3000	0.0002	Online
[46]	2018	0	0	Long Short-Term Memory (LSTM) RNNs	N/I	Xilinx Zynq-7045	769	N/I	Offline
[39]	2018	1	1	quadrature spline wavelet (QSW), PCA	N/I	FPGA cyclone II	0.145	0.806	Offline
[15]	2014	1	0	FIR, DWT, PSD, AR, Filter bank, Zero-crossing Histogram, Correlation, Phase synchronization, Mann–Whitney test, LSSVM	96.93	Spartan FPGA with a XC3S500E-PQ208	277.74	N/I	Online

**Table 3:** Summary of related works on embedded BCIs for functional substitution: N/I: No indication, 0: bad, 1: good.

Work	Year	$C_{s}$	$A_{d}$	Algorithms	Accuracy (%)	Platform	Time (ms)	Power (W)	Online/ Offline
[13]	2008	0	0	Average filter, PCA, Linear regression	74.6	Virtex 6	2420	1.11	Online
[49]	2010	1	0	FIR, DWT, SVM	N/I	Compact-RIO	N/I	12.81	Offline
[51]	2015	1	0	Theta spectra, threshold	71	FPGA Mobile tablet	N/I	4	Online
[52]	2010	1	0	STFFT, ICA, threshold	78.24	DSP	N/I	1.11	Online
[53]	2017	0	0	FFT, threshold	99.4	Micro2440SDK	∼8500	24	Online
[54]	2013	1	0	IIR, DWT, threshold hierarchical model	91	CompactDAQ	2	12	Online
[55]	2016	0	1	DWT, SVM	82.1	Odroid-xu4	0.11	20	Offline
[56]	2017	0	0	Band-pass filter, average power, temporal correlation	70	Arduino Due MCU	2.23	1	Offline
[57]	2014	0	0	FFT, SLIC	70	Micro2440 (ARM)	0.1	24	Offline
[58]	2017	0	1	ERD/ERS, Adaptive Threshold	75	Zynq ZC7030	402	4.12	Online, offline
[59]	2014	0	1	FFT, Mahalanobis distance	82	Blackfin, DSP	N/I	4.02	Online
[60]	2014	0	0	FFT	98.8	AT89S51, Tablet (AUSU)	42	12	Online
[48]	2010	0	0	Phase coding, FFT	89.29	Cyclone EP2C20Q FPG	30.14	∼27	Online
[61]	2012	1	0	FFT, Mardia test, Mahalanobis distance (MD)	77.6	Cyclone EP2C20Q FPG	30.14	∼27	Online
[14]	2012	1	0	Threshold	61.6	iPhone	32	∼6	Online
[62]	2012	0	0	FFT, Morlet Continuous Wavelet, Threshold	N/I	Spartan3 XC3S1400AN	1	N/I	Online
[63]	2011	0	0	PSD, LDA	73.58	ASIC, MSP430F1611, NRF24L	200	0.001395	Online
[23]	2017	1	1	Adaptive filter, CSP, MD	94.47	Stratix-IV	394	1.067	Offline
[64]	2018	1	1	WOLA filter bank, CSP, MD	80.2	Stratix-IV	430	0.67	Online, Offline
[65]	2012	0	0	Forward Filter, FLDA	73.96	Spartan 3E FPGA	N/I	N/I	Online
[66]	2012	0	0	FIR filterbank, Hidden Markov Models	76.5	Spartan 6 FPGA	N/I	N/I	Online
[67]	2016	1	0	adaptive filtering	N/I	FPGA Virtex-5 LX50T	N/I	N/I	Online
[68]	2020	0	0	PSD, band-pass filtering, canonical correlation analysis (CCA)	80	XC7K325T-2FFG900C	N/I	N/I	Online
[69]	2017	0	0	Median, FIR filter, FLDA	N/I	Virtex-5	0.01	0.67	Online
[70]	2015	0	0	ICA, Canonical Correlation Analysis (CCA)	86.5	FPGA	32000	N/I	Online
[71]	2016	0	0	Blind Source Separation (BSS), CCA	93.41	FPGA	N/I	N/I	Online
[72]	2018	0	0	Sparse Bayesian Learning (BSBL), multi-layer perceptron regressor	89.85	Virtex7, ARM	N/I	N/I	Online
[47]	2019	0	1	CNN	80.5	Xilinx BNN-PYNQ	1.97	0.025	Offline
[73]	2015	0	0	FFT, Threshold	88.88	Xilinx & PC Tablet	4430	70	Online
[74]	2018	1	0	Surface Laplacian, Separable Common Spatio Spectral Pattern (SCSSP), Mutual Information (MI), Linear Discriminant Analysis (LDA), and Support Vector Machine (SVM)	81.9	Virtex-6 FPGA	0.550	83	Online & offline
[75]	2020	0	0	FIR filter, averaging method	90.62	Cyclone II EP2C35 DSP	∼2000	∼27	Online
[76]	2019	1	0	Channel selection, wavelet, energy normalization, LDA	80	Xilinx	7.5	0.102	Offline
[77]	2013	0	0	FFT, threshold	92.5	FPGA, MCP3201 microcontroller	5200	1.74	Online
[78]	2020	0	0	Canonical correlation analysis (CCA)	76	DE0-nano board	0.00052	∼0.05	Online
[79]	2019	0	0	CCA	87.89	Cyclone IV EP4CE115	1500	∼0.05	Offline
[80]	2020	0	0	Long Short-Term Memory (LSTM)	87.89	MindReading photonic ULQ	1500	0.2155	Offline
[81]	2019	0	0	bandpass Butterworth filter, DWT, Feedforward Neural Network (FFNN)	96.09	Raspberry Pi 3B	N/I	5.77	Online

**Table 4:** Parameters of the evaluation criteria $E_{c}$.

Parameter	Value	Reason
ITR	Computed according to the equation (Equation (1)).	The ITR takes into consideration the system accuracy and the timing, which represent two criteria from the predefined criteria and are important to evaluate the EBCI systems.
α	=1/3, if the EBCI system is controlled by the evoked potential signals.	The EBCI system is more comfortable when it is controlled by SP. Thus, we are given the highest weight for the EBCI system controlled by SP.
	=2/3, if the EBCI system controlled by the spontaneous signals (SP).	
β	=1/3, if the EBCI system is static (same parameters for all subjects).	The EBCI system is more accurate when the EBCI parameters are defined for each subject. Thus, the highest weight is given for the EBCI system toke in consideration the inter-subject variability.
	=2/3, if the EBCI is adaptive.	
γ	=1/3, if the EBCI system is checked and validated according to the offline approach only.	The accuracy of the EBCI system is validated according to the online approach and is more reliable which reflects the usefulness of the EBCI system.
	=2/3, if the EBCI system is checked and validated according to the online approach.	
$P_{w}$	The measured power of the EBCI system.	One of the important criteria to evaluate the EBCI systems.
$N_{ch}$	Number of channels that is used during the recording of the brain signals.	The number of channels differ from one system to another and has an effect on the runtime and the power consumption. For this reason, it should be taken into consideration during the comparison between EBCIs.

## Data Availability

Not applicable.
